# 1-Bromo-2,3,5,6-tetra­fluoro-4-nitro­benzene

**DOI:** 10.1107/S160053681102201X

**Published:** 2011-06-18

**Authors:** Mario Stein, Anke Schwarzer, Jürg Hulliger, Edwin Weber

**Affiliations:** a Institut für Organische Chemie, TU Bergakademie Freiberg, Leipziger Strasse 29, D-09596 Freiberg/Sachsen, Germany; b Institut für Anorganische Chemie, TU Bergakademie Freiberg, Leipziger Strasse 29, D-09596 Freiberg/Sachsen, Germany; c Department of Chemistry and Biochemistry, University of Berne, Freiestrasse 3, CH-3012 Berne, Switzerland

## Abstract

In the title compound, C_6_BrF_4_NO_2_, the nitro group is twisted by 41.7 (3)° with reference to the arene ring mean plane. The main inter­actions stabilizing the crystal structure include O⋯Br contacts [3.150 (2) and 3.201 (2) Å], while F⋯F inter­actions are minor [2.863 (3)–2.908 (3) Å].

## Related literature

For halogen inter­actions in mol­ecular crystal structures, see: Awwadi *et al.* (2006 ▶); Brammer *et al.* (2001 ▶); Metrangolo *et al.* (2008 ▶). For fluorine-involved inter­actions, see: Schwarzer *et al.* (2010 ▶); Merz & Vasylyeva (2010 ▶); Schwarzer & Weber (2008 ▶); Reichenbächer *et al.* (2005 ▶). For the synthesis, see: Shtark & Shteingarts (1976 ▶).
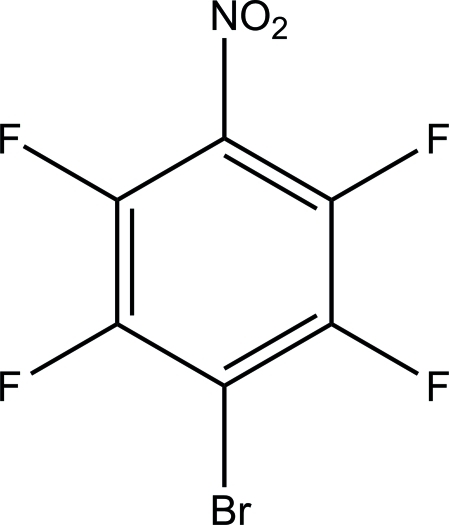


## Experimental

### 

#### Crystal data

C_6_BrF_4_NO_2_
*M* *_r_* = 273.98
Orthorhombic, 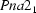

*a* = 5.6718 (3) Å
*b* = 10.9476 (6) Å
*c* = 12.2652 (8) Å
*V* = 761.58 (8) Å^3^
*Z* = 4
Mo *K*α radiation
μ = 5.44 mm^−1^
*T* = 93 K
0.13 × 0.13 × 0.10 mm

#### Data collection

Bruker SMART CCD area-detector diffractometer
Absorption correction: multi-scan (*SADABS*; Bruker, 2007 ▶) *T*_min_ = 0.536, *T*_max_ = 0.612
4314 measured reflections
1550 independent reflections
1447 reflections with *I* > 2σ(*I*)
*R*_int_ = 0.026

#### Refinement

*R*[*F* ^2^ > 2σ(*F* ^2^)] = 0.023
*wR*(*F* ^2^) = 0.053
*S* = 1.00
1550 reflections
127 parameters
1 restraint
Δρ_max_ = 0.32 e Å^−3^
Δρ_min_ = −0.53 e Å^−3^
Absolute structure: Flack (1983) ▶, 636 Friedel pairs
Flack parameter: 0.026 (10)

### 

Data collection: *SMART* (Bruker, 2007 ▶); cell refinement: *SAINT* (Bruker, 2007 ▶); data reduction: *SAINT*; program(s) used to solve structure: *SHELXS97* (Sheldrick, 2008 ▶); program(s) used to refine structure: *SHELXL97* (Sheldrick, 2008 ▶); molecular graphics: *SHELXTL* (Sheldrick, 2008 ▶); software used to prepare material for publication: *SHELXTL*.

## Supplementary Material

Crystal structure: contains datablock(s) global, I. DOI: 10.1107/S160053681102201X/su2278sup1.cif

Structure factors: contains datablock(s) I. DOI: 10.1107/S160053681102201X/su2278Isup2.hkl

Supplementary material file. DOI: 10.1107/S160053681102201X/su2278Isup3.cml

Additional supplementary materials:  crystallographic information; 3D view; checkCIF report

## Figures and Tables

**Fig. 1.**
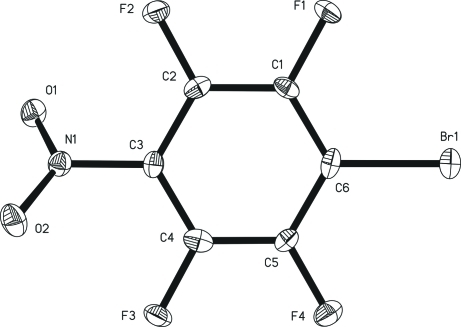
A view of the molecular structure of the title molecule, with the atom numbering and showing 50% probability displacement ellipsoids.

**Fig. 2.**
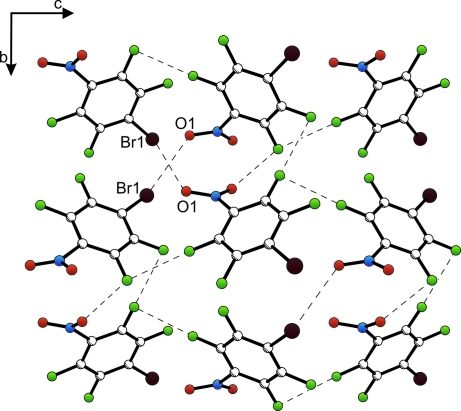
A partial view, along the a axis, of the crystal packing of the title compound. The O···Br, and potential O···F and F···F contacts are shown as broken lines (see Table 1 for details).

## supplementary crystallographic information

### Comment

Halogen interactions in molecular crystal structures have been the subject of a numer of studies (Awwadi *et al.*, 2006; Brammer *et al.*, 2001; Metrangolo *et al.*, 2008). Fluorine involved interactions in particular have been studied by us and others (Schwarzer *et al.*, 2010; Schwarzer & Weber, 2008; Reichenbächer *et al.*, 2005; Merz & Vasylyeva, 2010). In continuation of that work we report herein on the crystal structure of the title tetrafluoro-benzene compound.

In the title compound (Fig. 1) the plane of the nitro group (O1—N1—O2) shows a twist of 41.68 (28)° with reference to the phenyl ring, owing to repulsive interactions between the *ortho*-positioned fluorine (F2 and F3) and oxygen atoms. The N—O bond lengths are different (O1—N1: 1.217 (4) Å; O2—N1: 1.234 (3) Å) as a result of different intermolecular interactions.

In the crystal oxygen O1 is involved in two strong intermolecular contacts to bromine Br1 [3.150 (2) and 3.201 (2) Å], giving rise to the formation of a three-dimensional molecular network (Table 1 and Fig. 2). On the other hand, atom O2 forms a weak contact to atom F3 [2.823 (3) Å].

The fluorine···fluorine contacts [2.863 (3) – 2.908 (3) Å] are close to the sum of their van-der-Waals radii hence, they do not contribute significantly to the stabilization of the crystal packing. Moreover, there is no indication for the presence of either π^F^···π^F^ stacking or C—*X*···π^F^ interactions (*X* = O, F, Br). Hence, except for the O···Br interactions, the crystal structure is mostly determined by maximum symmetry and close-packing principles, which is reflected in the low melting point of 321 K.

### Experimental

The title compound was synthesized according to the published procedure (Shtark & Shteingarts, 1976). 3-bromo-1,2,4,5-tetrafluorobenzene (2.80 g, 12 mmol) and NO_2_BF_4_ (6.45 g, 48 mmol) were dissolved in 45 ml of sulfolan and stirred for 2 h at 338 K. After cooling the solution to room temperature, 120 ml water was added and the phases separated. The aqueous layer was extracted with chloroform (3 × 50 ml), dried (Na_2_SO_4_) and evaporated under reduced pressure. The crude product was purified by water steam distillation to yield 2.66 g (81%) of the title compound. Sublimation techniques yielded single crystals suitable for X-ray crystallography.

### Crystal data

**Table:**

C_6_BrF_4_NO_2_	*F*(000) = 520
*M**_r_* = 273.98	*D*_x_ = 2.390 Mg m^−^^3^
Orthorhombic, *P**n**a*2_1_	Mo *K*α radiation, λ = 0.71073 Å
Hall symbol: P 2c -2n	Cell parameters from 2319 reflections
*a* = 5.6718 (3) Å	θ = 3.7–32.8°
*b* = 10.9476 (6) Å	µ = 5.44 mm^−^^1^
*c* = 12.2652 (8) Å	*T* = 93 K
*V* = 761.58 (8) Å^3^	Needle, colourless
*Z* = 4	0.13 × 0.13 × 0.10 mm

### Data collection

**Table:**

Bruker SMART CCD area-detector diffractometer	1550 independent reflections
Radiation source: fine-focus sealed tube	1447 reflections with *I* > 2σ(*I*)
graphite	*R*_int_ = 0.026
phi and ω scans	θ_max_ = 27.5°, θ_min_ = 2.5°
Absorption correction: multi-scan (*SADABS*; Bruker, 2007)	*h* = −7→7
*T*_min_ = 0.536, *T*_max_ = 0.612	*k* = −14→12
4314 measured reflections	*l* = −15→15

### Refinement

**Table:**

Refinement on *F*^2^	Primary atom site location: structure-invariant direct methods
Least-squares matrix: full	Secondary atom site location: difference Fourier map
*R*[*F*^2^ > 2σ(*F*^2^)] = 0.023	*w* = 1/[σ^2^(*F*_o_^2^) + (0.0208*P*)^2^] where *P* = (*F*_o_^2^ + 2*F*_c_^2^)/3
*wR*(*F*^2^) = 0.053	(Δ/σ)_max_ = 0.008
*S* = 1.00	Δρ_max_ = 0.32 e Å^−^^3^
1550 reflections	Δρ_min_ = −0.53 e Å^−^^3^
127 parameters	Absolute structure: Flack (1983), 636 Friedel pairs
1 restraint	Flack parameter: 0.026 (10)

### Special details

**Table:**

Geometry. All e.s.d.'s (except the e.s.d. in the dihedral angle between two l.s. planes) are estimated using the full covariance matrix. The cell e.s.d.'s are taken into account individually in the estimation of e.s.d.'s in distances, angles and torsion angles; correlations between e.s.d.'s in cell parameters are only used when they are defined by crystal symmetry. An approximate (isotropic) treatment of cell e.s.d.'s is used for estimating e.s.d.'s involving l.s. planes.
Refinement. Refinement of *F*^2^ against ALL reflections. The weighted *R*-factor *wR* and goodness of fit *S* are based on *F*^2^, conventional *R*-factors *R* are based on *F*, with *F* set to zero for negative *F*^2^. The threshold expression of *F*^2^ > σ(*F*^2^) is used only for calculating *R*-factors(gt) *etc*. and is not relevant to the choice of reflections for refinement. *R*-factors based on *F*^2^ are statistically about twice as large as those based on *F*, and *R*- factors based on ALL data will be even larger.

### Fractional atomic coordinates and isotropic or equivalent isotropic displacement parameters (Å^2^)

**Table:**

	*x*	*y*	*z*	*U*_iso_*/*U*_eq_	
Br1	1.16169 (4)	0.62891 (2)	0.12241 (5)	0.01760 (10)	
F1	0.9560 (3)	0.69267 (15)	−0.10033 (17)	0.0188 (4)	
F2	0.6066 (3)	0.58458 (16)	−0.20909 (18)	0.0178 (4)	
F3	0.5004 (3)	0.30738 (15)	0.08418 (15)	0.0181 (4)	
F4	0.8565 (3)	0.41466 (18)	0.19058 (19)	0.0192 (4)	
N1	0.3610 (4)	0.3813 (2)	−0.1276 (3)	0.0148 (6)	
O1	0.3925 (4)	0.36408 (18)	−0.2245 (2)	0.0181 (5)	
O2	0.1833 (3)	0.3518 (2)	−0.0760 (2)	0.0207 (6)	
C1	0.8465 (4)	0.5971 (3)	−0.0542 (3)	0.0134 (7)	
C2	0.6671 (4)	0.5405 (3)	−0.1118 (3)	0.0136 (7)	
C3	0.5498 (4)	0.4424 (3)	−0.0658 (3)	0.0128 (7)	
C4	0.6105 (5)	0.4023 (3)	0.0374 (3)	0.0146 (7)	
C5	0.7936 (4)	0.4574 (3)	0.0933 (3)	0.0129 (8)	
C6	0.9119 (4)	0.5558 (3)	0.0468 (3)	0.0146 (7)	

### Atomic displacement parameters (Å^2^)

**Table:**

	*U*^11^	*U*^22^	*U*^33^	*U*^12^	*U*^13^	*U*^23^
Br1	0.01516 (11)	0.01875 (15)	0.01890 (19)	−0.00140 (8)	−0.00323 (18)	−0.0046 (2)
F1	0.0214 (7)	0.0153 (9)	0.0198 (13)	−0.0066 (6)	0.0011 (8)	0.0038 (8)
F2	0.0241 (8)	0.0159 (9)	0.0135 (13)	−0.0005 (7)	−0.0031 (9)	0.0027 (9)
F3	0.0224 (7)	0.0155 (9)	0.0164 (12)	−0.0057 (6)	0.0040 (8)	0.0017 (7)
F4	0.0256 (9)	0.0177 (10)	0.0142 (14)	0.0018 (7)	−0.0024 (8)	0.0009 (9)
N1	0.0150 (12)	0.0158 (15)	0.014 (2)	−0.0005 (8)	−0.0002 (11)	−0.0015 (13)
O1	0.0205 (8)	0.0206 (12)	0.0133 (16)	−0.0014 (7)	−0.0005 (10)	−0.0048 (10)
O2	0.0134 (9)	0.0245 (14)	0.0241 (17)	−0.0025 (7)	0.0025 (10)	−0.0016 (11)
C1	0.0165 (12)	0.0082 (15)	0.016 (2)	−0.0009 (10)	0.0038 (13)	0.0006 (13)
C2	0.0160 (12)	0.0125 (16)	0.012 (2)	0.0048 (9)	0.0001 (12)	−0.0004 (14)
C3	0.0099 (10)	0.0143 (15)	0.014 (2)	0.0018 (10)	0.0001 (12)	−0.0040 (13)
C4	0.0169 (12)	0.0107 (15)	0.016 (2)	0.0019 (10)	0.0045 (13)	0.0003 (13)
C5	0.0150 (10)	0.0158 (15)	0.008 (2)	0.0030 (9)	0.0001 (11)	−0.0003 (12)
C6	0.0110 (10)	0.0169 (15)	0.016 (2)	0.0014 (10)	−0.0020 (12)	−0.0065 (13)

### Geometric parameters (Å, °)

**Table:**

Br1—C6	1.874 (3)	F4—F3^iii^	2.877 (2)
Br1—O1^i^	3.150 (2)	F4—F2^vii^	2.901 (2)
Br1—O1^ii^	3.201 (2)	N1—O1	1.217 (4)
F3—O2^iii^	2.823 (3)	N1—O2	1.234 (3)
F1—C1	1.342 (3)	N1—C3	1.472 (4)
F1—F2^iv^	2.908 (3)	O1—Br1^ix^	3.150 (2)
F2—C2	1.332 (4)	O1—Br1^x^	3.201 (2)
F2—F3^v^	2.863 (3)	O2—F3^viii^	2.823 (3)
F2—F4^v^	2.901 (2)	C1—C6	1.369 (5)
F2—F1^vi^	2.908 (3)	C1—C2	1.385 (4)
F3—C4	1.341 (3)	C2—C3	1.385 (4)
F3—F2^vii^	2.863 (3)	C3—C4	1.383 (5)
F3—F4^viii^	2.877 (2)	C4—C5	1.382 (4)
F4—C5	1.331 (4)	C5—C6	1.391 (4)
			
C6—Br1—O1^i^	155.87 (10)	N1—O1—Br1^ix^	134.13 (18)
C6—Br1—O1^ii^	124.16 (9)	N1—O1—Br1^x^	133.10 (18)
O1^i^—Br1—O1^ii^	73.03 (6)	Br1^ix^—O1—Br1^x^	75.36 (6)
C1—F1—F2^iv^	169.53 (15)	N1—O2—F3^viii^	144.98 (19)
C2—F2—F3^v^	176.03 (17)	F1—C1—C6	120.9 (3)
C2—F2—F4^v^	127.90 (16)	F1—C1—C2	118.2 (3)
F3^v^—F2—F4^v^	55.33 (6)	C6—C1—C2	120.8 (3)
C2—F2—F1^vi^	88.19 (17)	F2—C2—C3	121.5 (3)
F3^v^—F2—F1^vi^	89.86 (7)	F2—C2—C1	119.0 (3)
F4^v^—F2—F1^vi^	85.76 (7)	C3—C2—C1	119.5 (3)
C4—F3—O2^iii^	90.56 (18)	C4—C3—C2	120.0 (3)
C4—F3—F2^vii^	99.04 (18)	C4—C3—N1	120.6 (3)
O2^iii^—F3—F2^vii^	161.63 (9)	C2—C3—N1	119.5 (3)
C4—F3—F4^viii^	168.70 (16)	F3—C4—C5	118.4 (3)
O2^iii^—F3—F4^viii^	84.17 (8)	F3—C4—C3	121.4 (3)
F2^vii^—F3—F4^viii^	83.52 (8)	C5—C4—C3	120.1 (3)
C5—F4—F3^iii^	88.06 (17)	F4—C5—C4	119.5 (3)
C5—F4—F2^vii^	97.86 (13)	F4—C5—C6	120.7 (3)
F3^iii^—F4—F2^vii^	116.85 (8)	C4—C5—C6	119.8 (3)
O1—N1—O2	125.5 (3)	C1—C6—C5	119.7 (3)
O1—N1—C3	117.8 (2)	C1—C6—Br1	120.8 (2)
O2—N1—C3	116.6 (3)	C5—C6—Br1	119.5 (2)
			
O2—N1—O1—Br1^ix^	−164.81 (19)	F4^viii^—F3—C4—C5	−50.3 (12)
C3—N1—O1—Br1^ix^	15.2 (4)	O2^iii^—F3—C4—C3	65.5 (3)
O2—N1—O1—Br1^x^	−49.6 (4)	F2^vii^—F3—C4—C3	−130.3 (3)
C3—N1—O1—Br1^x^	130.4 (2)	F4^viii^—F3—C4—C3	127.4 (9)
O1—N1—O2—F3^viii^	110.3 (4)	C2—C3—C4—F3	−179.9 (3)
C3—N1—O2—F3^viii^	−69.7 (4)	N1—C3—C4—F3	0.0 (4)
F2^iv^—F1—C1—C6	−133.4 (10)	C2—C3—C4—C5	−2.2 (4)
F2^iv^—F1—C1—C2	47.2 (13)	N1—C3—C4—C5	177.7 (3)
F3^v^—F2—C2—C3	174 (2)	F3^iii^—F4—C5—C4	65.3 (3)
F4^v^—F2—C2—C3	30.2 (4)	F2^vii^—F4—C5—C4	−51.5 (3)
F1^vi^—F2—C2—C3	113.4 (3)	F3^iii^—F4—C5—C6	−113.9 (3)
F3^v^—F2—C2—C1	−4(3)	F2^vii^—F4—C5—C6	129.2 (2)
F4^v^—F2—C2—C1	−147.6 (2)	F3—C4—C5—F4	0.5 (4)
F1^vi^—F2—C2—C1	−64.4 (3)	C3—C4—C5—F4	−177.2 (2)
F1—C1—C2—F2	−1.5 (4)	F3—C4—C5—C6	179.8 (3)
C6—C1—C2—F2	179.1 (3)	C3—C4—C5—C6	2.0 (4)
F1—C1—C2—C3	−179.4 (3)	F1—C1—C6—C5	179.2 (3)
C6—C1—C2—C3	1.2 (4)	C2—C1—C6—C5	−1.4 (4)
F2—C2—C3—C4	−177.2 (3)	F1—C1—C6—Br1	−1.6 (4)
C1—C2—C3—C4	0.6 (4)	C2—C1—C6—Br1	177.8 (2)
F2—C2—C3—N1	2.9 (4)	F4—C5—C6—C1	179.0 (3)
C1—C2—C3—N1	−179.2 (3)	C4—C5—C6—C1	−0.2 (4)
O1—N1—C3—C4	−138.6 (3)	F4—C5—C6—Br1	−0.2 (4)
O2—N1—C3—C4	41.4 (4)	C4—C5—C6—Br1	−179.4 (2)
O1—N1—C3—C2	41.3 (4)	O1^i^—Br1—C6—C1	−143.2 (2)
O2—N1—C3—C2	−138.7 (3)	O1^ii^—Br1—C6—C1	86.1 (3)
O2^iii^—F3—C4—C5	−112.3 (3)	O1^i^—Br1—C6—C5	36.0 (4)
F2^vii^—F3—C4—C5	52.0 (3)	O1^ii^—Br1—C6—C5	−94.7 (2)

Symmetry codes: (i) −*x*+2, −*y*+1, *z*+1/2; (ii) −*x*+3/2, *y*+1/2, *z*+1/2; (iii) *x*+1/2, −*y*+1/2, *z*; (iv) *x*+1/2, −*y*+3/2, *z*; (v) −*x*+1, −*y*+1, *z*−1/2; (vi) *x*−1/2, −*y*+3/2, *z*; (vii) −*x*+1, −*y*+1, *z*+1/2; (viii) *x*−1/2, −*y*+1/2, *z*; (ix) −*x*+2, −*y*+1, *z*−1/2; (x) −*x*+3/2, *y*−1/2, *z*−1/2.
